# Africanized honey bees in Colombia exhibit high prevalence but low level of infestation of *Varroa* mites and low prevalence of pathogenic viruses

**DOI:** 10.1371/journal.pone.0244906

**Published:** 2021-05-20

**Authors:** Víctor Manuel Tibatá, Andrés Sanchez, Evan Palmer-Young, Howard Junca, Victor Manuel Solarte, Shayne Madella, Fernando Ariza, Judith Figueroa, Miguel Corona

**Affiliations:** 1 Facultad de Medicina Veterinaria y Zootecnia, Grupos de Investigación AYNI–Ciencia y Tecnología Apícola, Universidad Nacional de Colombia, Bogotá, Colombia; 2 Bee Research Lab, United States Department of Agriculture, Beltsville, MD, United States of America; 3 RG Microbial Ecology, Div. Ecogenomics & Holobionts–Microbiomas Foundation, Chia, Colombia; Universitat Leipzig, GERMANY

## Abstract

The global spread of the ectoparasitic mite *Varroa destructor* has promoted the spread and virulence of highly infectious honey bee viruses. This phenomenon is considered the leading cause for the increased number of colony losses experienced by the mite-susceptible European honey bee populations in the Northern hemisphere. Most of the honey bee populations in Central and South America are Africanized honey bees (AHBs), which are considered more resistant to *Varroa* compared to European honey bees. However, the relationship between *Varroa* levels and the spread of honey bee viruses in AHBs remains unknown. In this study, we determined *Varroa* prevalence and infestation levels as well as the prevalence of seven major honey bee viruses in AHBs from three regions of Colombia. We found that although *Varroa* exhibited high prevalence (92%), its infestation levels were low (4.5%) considering that these populations never received acaricide treatments. We also detected four viruses in the three regions analyzed, but all colonies were asymptomatic, and virus prevalence was considerably lower than those found in other countries with higher rates of mite-associated colony loss (DWV 19.88%, BQCV 17.39%, SBV 23.4%, ABPV 10.56%). Our findings indicate that AHBs possess a natural resistance to *Varroa* that does not prevent the spread of this parasite among their population, but restrains mite population growth and suppresses the prevalence and pathogenicity of mite-associated viruses.

## Introduction

Colombian beekeepers have primarily used AHBs derived from *A*. *mellifera scutellata* since they arrived in Colombia in 1979 [1,2] after originating in Brazil in 1956 [3]. However, many local beekeepers abandoned this practice after the arrival of AHBs, mainly due to their higher defensiveness. Subsequent generations of Colombian beekeepers adapted their management techniques to deal with the defensiveness of AHBs and at the same time, take advantage of their positive characteristics, including noticeably increased resistance to *Varroa destructor* infestation [4,5]. This ectoparasite arrived in Colombia in the 1980s [6] and spread throughout all continental territories, excluding the isolated San Andrés islands located in the Atlantic Ocean.

Honey bee populations in the Northern hemisphere have experienced severe losses in recent years [7]. These losses have been primarily attributed to infestations by the ectoparasitic mite *V*. *destructor* [8,9]. In addition to the direct harmful effects on honey bee health, *V*. *destructor* is an effective vector for several pathogenic honey bee viruses [10–12] especially deformed wing virus (DWV), that play a crucial role in colony losses [8,9,11,13,14]. Currently, twenty-three known viral pathogens affect honey bees in different parts of the world [15]. Several of the most pathogenic of them, including DWV, sacbrood virus (SBV), black queen cell virus (BQCV), acute bee paralysis virus (ABPV), chronic bee paralysis virus (CBPV) and Israeli acute paralysis virus (IAPV) [16–19], have been found in South America [20–27]. Although low levels of viruses are common in honey bees in the absence of *Varroa* [10,28–31], infestation with this parasite enhances the transmission of viral infections and their pathogenicity [11,32–34].

At present, beekeepers in the Northern hemisphere—who maintain European-derived populations of honeybees—rely on miticides to control *Varroa* infestations and their associated viruses. In contrast, mite control treatments are seldom necessary in South American countries with predominant AHB populations [21,25,35–37] as well as in African countries with populations of *Apis mellifera scutellata* [38]. Results of studies obtained in specific geographic regions of South America and Mexico are consistent with the view that AHBs are more resistant to *Varroa* infestations compared with European honey bees (EHBs) [5,36,39–41]. However, this mite still affects AHB colony fitness as reflected by reduced production of honey [41,42].

The AHB populations in Colombia offer the opportunity to study a natural selection process where populations that are adapted to a tropical climate reproduce and thrive without treatments to control pathogens [43]. AHBs relative resistance to *Varroa* infestation might be expected to translate into lower levels of viral infection. However, the prevalence of viruses and their relationship to *Varroa* in AHBs remains inconclusive. For example, one study found no differences in viral prevalence between AHBs and EHBs [37], but another reported increased viral resistance in AHBs compared with EHBs [44]. These conflicting results highlight the need for large-scale field studies to elucidate the epidemiology of *V*. *destructor* prevalence and infestation levels and their relationship to viral infection in AHB populations, such as those found in Latin American countries [45]. In this study, we determined *V*. *destructor* infestation levels and the prevalence of seven major honey bee viruses in three regions of Colombia, using honey bee populations that we previously confirmed were composed exclusively of AHBs [43]. Our results enhance knowledge about the relationship between *V*. *destructor* and viruses in AHBs and provide the first large-scale field survey of honey bee parasites and pathogens in this altitudinally and seasonally varied equatorial country.

## Materials and methods

### Study design and sampling regions

**Fig 1:**
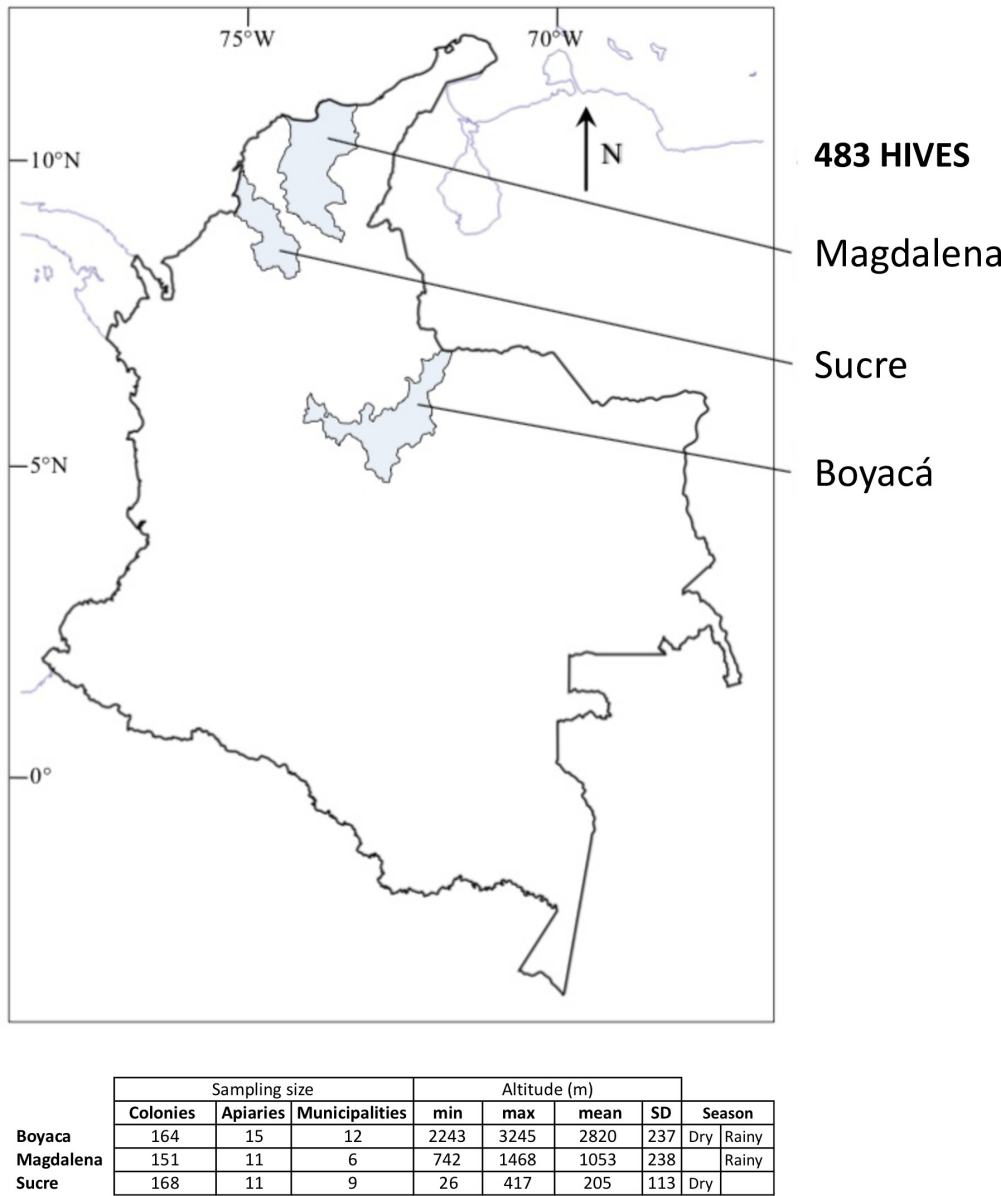
**Map of Colombia showing the geographic localization of the three regions analyzed in this study (highlighted in blue) and the number of stationary colonies sampled in each region.** Minimum (min), maximum (max), mean and standard deviation (SD) are indicated in meters (m).

This study was conducted in three geographical regions located in three representative beekeeping regions of Colombia: Magdalena, Sucre and Boyacá (Fig 1). All of the sampled colonies were stationary; none were migratory. The number of apiaries, colonies, and municipalities sampled were as it follows: In Magdalena, 151 colonies belonging to eleven apiaries located in six municipalities. In Sucre, 168 colonies from eleven apiaries located in nine municipalities. In Boyacá, 164 colonies from fifteen apiaries distributed throughout twelve counties. The apiaries resided in neotropical regions of Colombia which can have two distinct seasons per year. The “dry season” is characterized by moderate drought, whereas the “rainy season” accounts for most of the annual rainfall [46]. Collections were completed in the Magdalena region during the rainy season (November 2013 and June 2014) and the Sucre region during the dry season (March 2013 and March 2014). Sampling in the Boyacá region deserves special considerations: In the South, rainfalls exhibit a unimodal bi-seasonal pattern. In the North sub-region, pluvial precipitations present a bimodal tetra-seasonal pattern [46]. Thus, the timing of the rainy season varies across sub-regions. Samples in Boyacá were collected on the following dates, municipalities and corresponding weather season: October 2013, Soata, Turquemene, Umbita and Guacheta (rainy season); July 2014, San Mateo, Guacheta, Belem, Tutauza (dry season); July 2014, Rondon and Viracacha (rainy season). The study regions encompassed a wide range of altitudes, from near sea level to >3000 m, and ranged in climate from tropical at low altitude to temperate at high altitude. The apiaries in Sucre ranged from an altitude of 26–417 m with a mean of 205 m above the sea level and a tropical climate. Apiaries in Magdalena ranged from 742–1468 m, with a mean of 1053 m and an intermediate, subtropical climate. Apiaries in Boyacá ranged from 2243–3245 m (*±* 1002 m), with a mean of 2820 m and a temperate climate (S1 Dataset).

Across 5400 colonies from the three regions, 483 were randomly sampled. Sample size estimation was obtained by the program WinEpi from the University of Zaragoza (http://www.winepi.net/) using the formula for known population size (5400 colonies), with a confidence level of 97%, considering an expected minimum prevalence of 2% for each of each viral disease. This formula was used to determine *Varroa* prevalence and infestation levels as well as for sampling of adults and larvae for the determination of viral prevalence. Due to an absence of information about bee morbidity or mortality in the sampled regions, collections were performed randomly. In the sampled apiaries, no symptoms related to viral diseases were observed. Samples in which viruses were detected by RT-PCR were considered infected but not necessarily diseased.

### Determination of *Varroa* prevalence and infestation level

To determine the prevalence and infestation level of *V*. *destructor* in the 483 colonies, 200 adult bees from each colony were deposited in flasks containing 96% ethanol and then transported to the laboratory to obtain the *Varroa* infestation level (VIL) according to De Jong et al. [47]. Bees were collected inside the colony directly from frames located in the brood chamber to obtain a sample composed of mainly younger, nurse bees. The VIL was calculated as a percentage by dividing the number of *Varroa* mites by the number of bees and multiplied by 100.

### Collection of samples for analysis of RNA viruses

In each of the 483 selected colonies, two samples were taken: One consisted of 60 larvae and another with 60 adult bees (both taken in pools). The two samples were collected from a single frame of each hive. Larvae were taken directly from each hive and deposited in 50 mL conical tubes, which contained RNAlater (Qiagen) to avoid RNA degradation. Adult bees were collected alive inside the same hive and were euthanized by inhalation with ethyl acetate in a lethal chamber, according to international standards. All samples were stored and kept in liquid nitrogen until subsequent processing.

### RNA extraction and cDNA synthesis

#### Adults

Each pooled sample of 60 frozen adult bees was placed inside Ziploc bags, and 30 ml of lysis buffer was added (Guanidium thiocyanate 0.8M; ammonium thiocyanate 0,4M; sodium acetate 3M, glycerol 5% and Triton-X 100 2%). The material was smashed with a rolling pin. An aliquot of 620 μl of macerated tissue was taken from each pool, mixed with 320 μl of acid phenol (pH 4.0), incubated 10 min at 95°C and cooled in an ice bath. Then, 200 μl of chloroform was added. Subsequently, samples were vortexed and centrifuged at 12000 x g for 15 minutes at 4°C. The aqueous phase was extracted, and one volume of cold isopropanol was added, mixed and centrifuged at 12000xg for 15 minutes. The resulting pellet was washed with 75% ethanol, resuspended in 200 μl of RNase-free water and stored at -70°C.

#### Larvae

Each pooled sample of 60 larvae was macerated directly in the collection tube with disposable pestles. RNA was extracted with the High Pure Kit Nucleic Acid Kit DNA-RNA (Roche Diagnostics) according to the manufacturer’s recommendations. To determine the integrity and quality of extracted RNA, aliquots of randomly selected samples were quantified by fluorometry (Qubit 2.0 Invitrogen) and analyzed by electrophoresis on denaturing agarose gels. For each larval and adult sample, cDNA was synthesized using 500 μg of total RNA and reagents from the Transcriptor First Strand cDNA Synthesis kit (Roche Diagnostics), using the following thermal profile: 10 min., 25°C; 30 min., 55°C; 5 min., 85°C.

### Internal amplification control

Before performing the viral diagnosis, to ensure that lack of amplification was not due to poor extraction or the presence of PCR inhibitors, the samples were subjected to amplification of a 184 bp fragment of *Apis mellifera* Beta Actin gene, according to the protocol reported by Chen et al., (2005) [48], but adapted to a quantitative PCR protocol with SYBR green. The amplified fragment’s size was verified by electrophoresis on 2% agarose gel, in TAE1X, visualized under UV light on a transilluminator (NyxTechnik) and compared with GeneRuler ladder of 100–1000 bp (Thermo Scientific).

### Detection of viral pathogens by real-time PCR with SYBRGreen

We followed protocols reported by other authors to detect of the targeted viruses [48–54] (S1 Table). In this study, these protocols were adjusted to a real-time format with Green Essential FastStart Master kit (Roche Diagnostics), in a Nano LightCycler (Roche Diagnostics). Data were analyzed in the LightCycler SW 1.0 software. The results were interpreted as the absence or presence of the amplified product, without performing quantifications. PCR techniques were done in separate reactions for each virus (Single PCR) with an aliquot of the cDNA from each larval and adult bee samples. Positive controls consisted of PCR products cloned in plasmids, which were obtained from the USDA-ARS Bee Research Laboratory (Beltsville MD, USA) and the Entomology Department, Volcani Center (Bet Dagan, Israel). Negative controls consisted of ultrapure water. Positive viral detections were verified by temperature melting curves analysis. Amplified viral fragments had the following lengths and melting point temperatures: DWV 700 bp, 82°C [55]; ABPV, 452 bp, 80°C [49]; SBV 340 bp, 83.7 [51], BQCV 284 bp, 80.5°C [50]. Viral prevalence was defined as the ratio between the number of PCR-positive to the total number of (colony-level) samples.

### Determining the prevalence of virus

The sampling was designed to be a broad survey not only searching for specific disease symptoms but also the presence and absence of the virus. Therefore, the virus-positive samples obtained by RT-PCR were interpreted as infected, but it does not imply that the originating material showed bees with overt illness. The prevalence of each virus was defined as the ratio between: Number of infected colonies/total number of sampled colonies. For each virus, the prevalence was established in each region and the subsequent mean for all the three regions. We followed two criteria to determine a sample as positive: a) confirmation of a single peak of the melting curve and b) the correct size of the amplified fragment. Additionally, amplicons of positive samples were sequenced (Macrogen, Seoul, South Korea) and compared to NCBI database using Blast-n.

### Statistical analysis

Initial analyses of normality of the values were conducted using the Kolmogorov-Smirnov/Lilliefor test [56]. Analyses of VIL and viral prevalence were conducted using the Mann Whitney U test [57] and one-way ANOVA followed by Bonferroni correction, respectively. Correlations among viral prevalence, altitude and season were conducted using Spearman’s non-parametric correlation [57,58] and these values were used to construct a dissimilarity matrix (dissimilarity = 1—correlation coefficient). The analyses of VIL and viral prevalence were calculated per region using the colony as a unit. Because Magdalena was sampled only during the rainy season and Sucre only during the dry season, we tested seasonal effects within the Boyacá region only. Similarly, we analyzed the effects of altitude within each region to avoid confounding the altitude effects with those of season.

## Results

### Prevalence and infestation level of *Varroa destructor*

**Fig 2:**
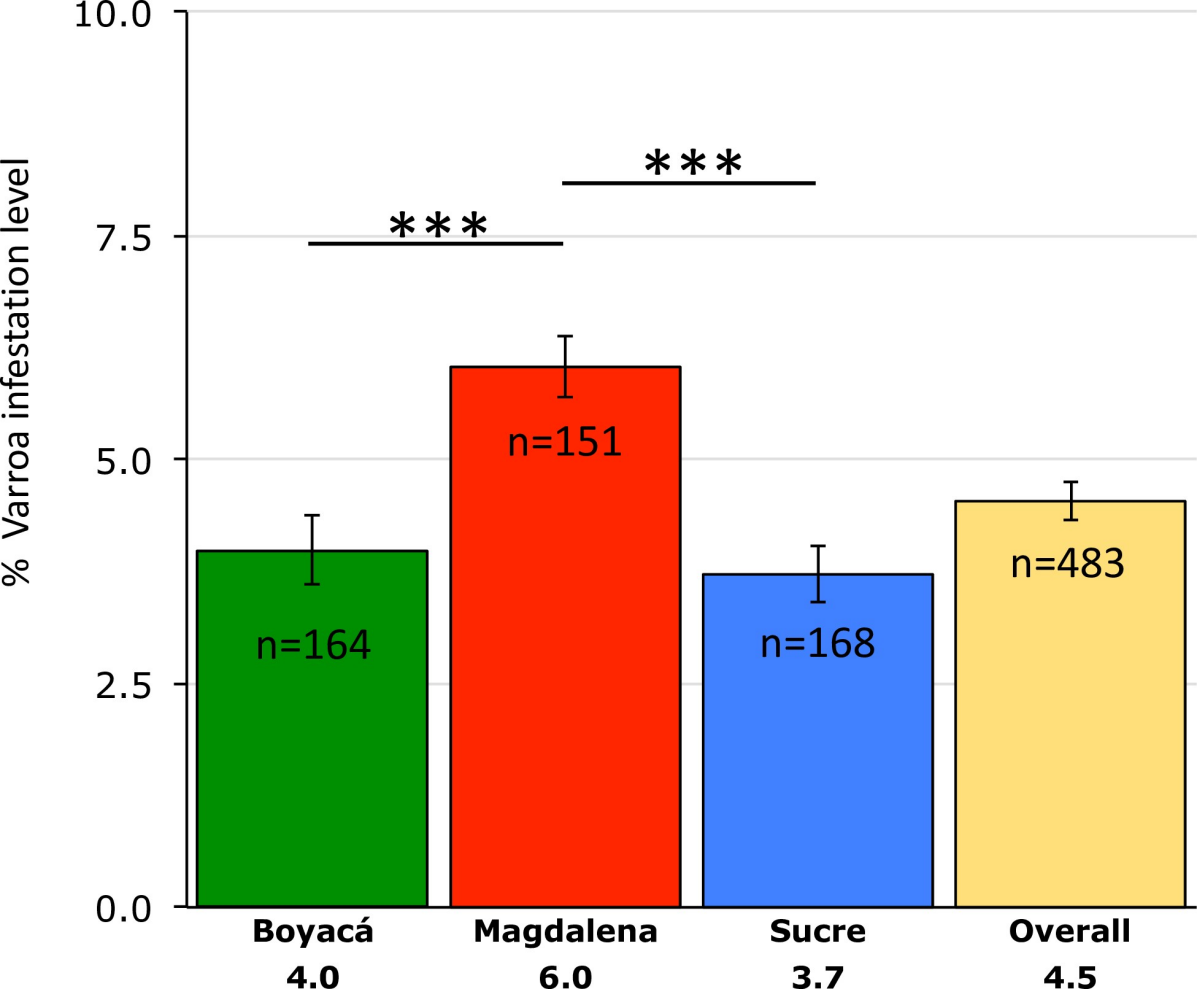
*Varroa* infestation levels per region. The y-axis indicates mean percentage of *Varroa* infestation. Error bars represent SE. Non-parametric data were analyzed with the Mann Whitney U test.

We found that the vast majority of the 483 colonies sampled were infested with *V*. *destructor* mites. Prevalence of *V*. *destructor* by region was as follows: Boyacá 89%, Magdalena 96% and Sucre 90%. *Varroa* infestation level (VIL) in Magdalena was higher than in Boyacá (U = 16,955, p = <0.001) and Sucre (U = 17,863, p = <0.001), but levels did not differ significantly between Boyacá and Sucre (U = 14,243, p = 0.59) (Fig 2). Overall, 68% of colonies showed VIL values below 5%, 20.6% had VIL between 5 to 10%, 9% of colonies presented VIL between 10 to 15% and only 2.4% registered infestations level above 15%. The region with the highest proportion of colonies with VIL above 5% was Magdalena. In contrast, Boyacá had a higher proportion of samples with VIL below 5% (S1 Fig). We found significantly higher VIL (Mann-Whitney U test p<0.001) in the samples collected during the rainy season (mean 5.0, SE 0.312) compared with the samples collected during the dry season (mean 4. 1, SE 0.271). However, the seasonal effects are confounded with regional and altitude effects, given that all samples from the relatively high-altitude Magdalena region were collected during the rainy season, whereas all samples from the low-altitude Sucre region were collected during the dry season.

### Viral co-infection

Of the seven viral pathogens tested, four of them (DWV, BQCV, SBV, ABPV) were found in both samples of larvae and adults from all three regions. The other three viruses tested (CBPV, IAPV, KBV) were not detected in any of the samples. Of the 483 colonies analyzed, we found no viruses in 35%, one virus in 31%, two viruses in 22.8%, three viruses in 10.1%, and all four viruses in only 1.1% of the colonies (S2 Table).

### Viral prevalence

**Fig 3:**
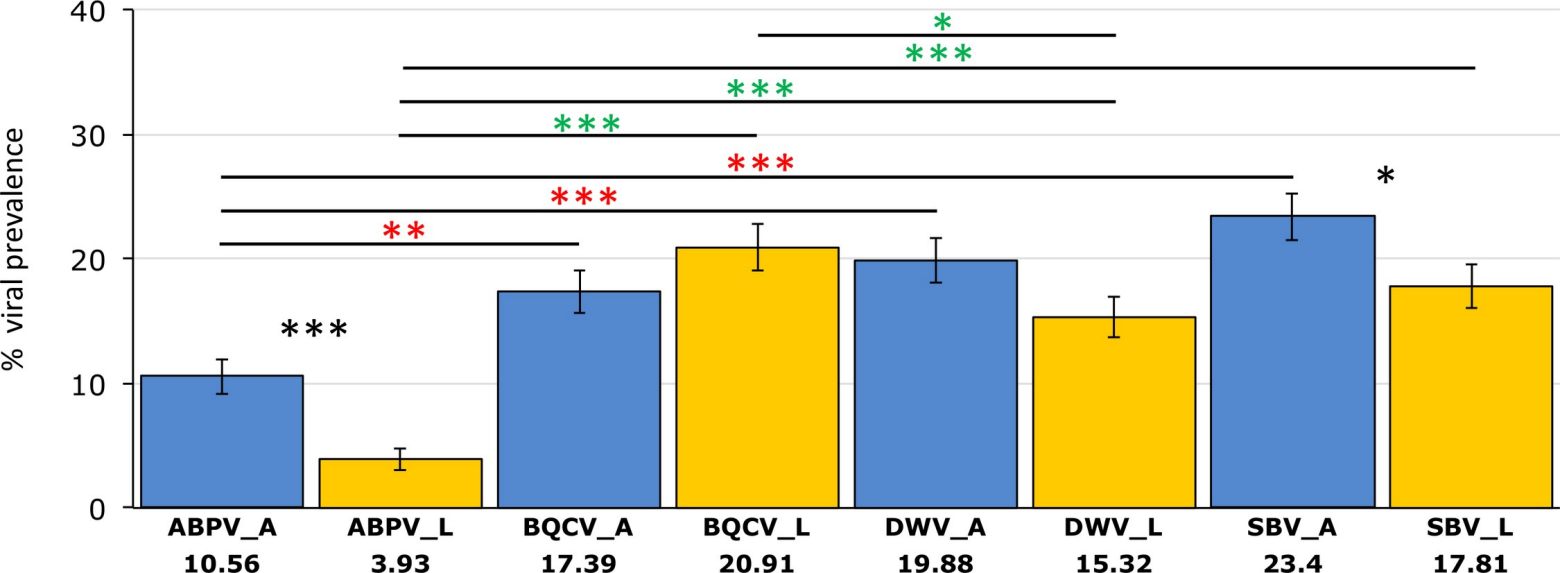
**Overall percentages of viral prevalence (y-axis) in adults (A, blue bars) and larvae (L, yellow bars).** N = 483. Significant differences in viral prevalence between larvae and adults are represented with black stars. Significant differences in the prevalence of the different viruses in adults and in larvae are represented by red and green starts, respectively (p< 0.05 *, p<0.001 **, p<0.0001***). Error bars represent SE. Data were analyzed using one-way ANOVA followed by Bonferroni correction.

In adults, SBV showed the highest percentage of prevalence (23.4%), followed by DWV (19.88%), BQCV (17.39%) and ABPV (10.56%). In larvae, BQCV was the virus with the higher percentage of prevalence (20.91%), followed by SBV (17.81%), DWV (15.32%) and ABPV (3.93%) (Fig 3, S3 Table). Comparison between adult and larvae, revealed similar trends in DWV and BQCV, where no significant differences in the prevalence of these viruses were observed between the two life stages. In contrast, significant differences were found between larvae and adult stages for ABPV (F = 16, p<0.0001) and SBV (F = 4.62, p = 0.031). In adults, significant differences among viral prevalence were observed among ABPV with BQCV (F = 9.45, p = 0.002), DWV (F = 16.49, p<0.001) and SBV (F = 29.02, p<0.001). In larvae, significant differences among viral prevalence were observed among ABPV with BQCV (F = 68.38, p<0.001), DWV (F = 37.3, p<0.001) and SBV (F = 50.37, p<0.001), and between BQCV with DWV (F = 5.1, p = 0.021).

**Fig 4:**
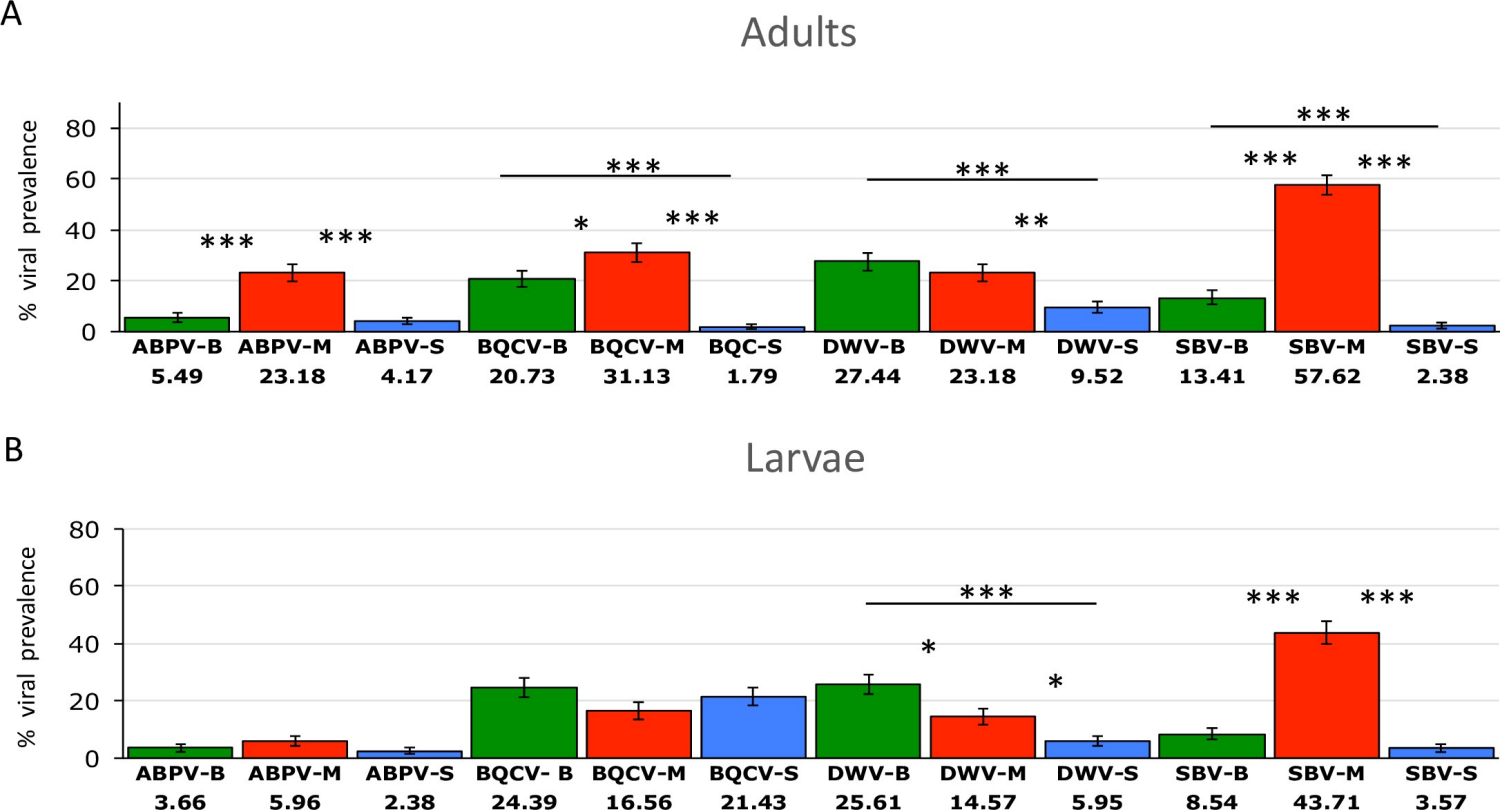
Regional percentages of viral prevalence in adults (panel A) and larvae (panel B). Boyacá (n = 165), Magdalena (n = 157) Sucre (n = 169). Names of the regions in the x-axis are abbreviated as follow: Boyacá (B, green bars), Magdalena (M, red bars) Sucre (S, blue bars). Error bars represent SE. Data were analyzed using one-way ANOVA followed by Bonferroni correction.

Differences among regions were most evident in adults (Fig 4). Samples from Magdalena had the highest prevalence of the three most frequently detected viruses, whereas samples from Sucre had the lowest levels. Significant differences were found in the prevalence of the following viruses between the following regions: Boyacá and Magdalena: ABPV (F = 21.75, p<0.001), BQCV (F = 4.48, p = 0.035) and SBV (F = 85.97, p<0.001). Magdalena and Sucre: ABPV (F = 27.12, p<0.001), BQCV (F = 61.45, p<0.001), DWV (F = 11.36, p<0.001) and SBV (F = 188.6, p<0.001). Boyacá and Sucre: BQCV (F = 32.88, p<0.001), DWV (F = 18.65, p<0.001) and SBV (F = 14.53, p<0.001) (Fig 4). In larvae, SBV showed a regional pattern similar to that observed in adults. However, prevalence of larval DWV was highest in Boyacá rather than in Magdalena, but likewise low in Sucre (Fig 4). Significant differences in larval viral prevalence between regions were as follows: Boyacá and Magdalena: DWV (F = 5.99, p = 0.015) and SBV (F = 60.93, p<0.001). Magdalena and Sucre: DWV (F = 6.64, p = 0.01) and SBV (F = 94.58, p<0.001). Boyacá and Sucre: DWV (F = 26.03, p<0.001).

### Correlations among variables

#### Correlations among viral prevalence

**Fig 5:**
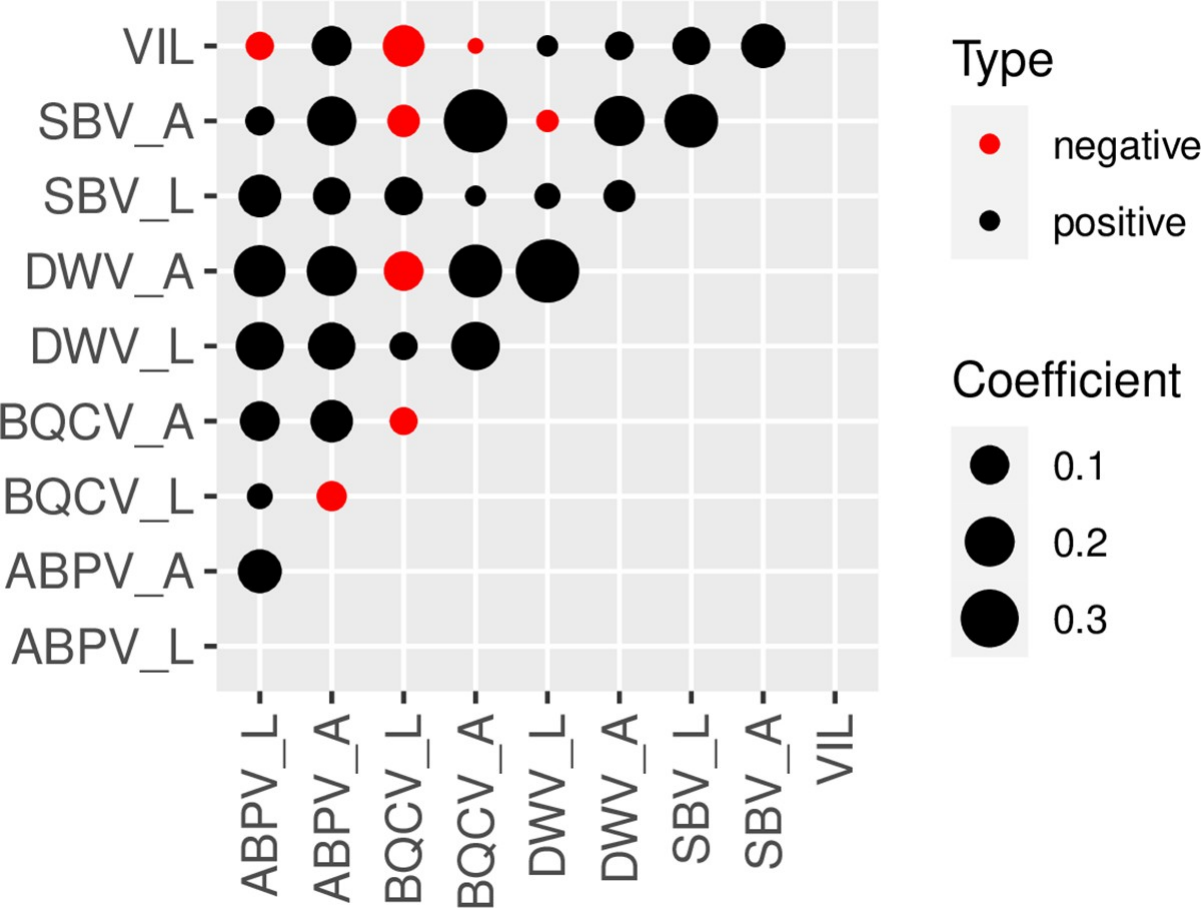
Correlations among *Varroa* infestation levels and viral prevalence in adults (A) and larvae (L). The circle size is proportional to the correlation coefficient. Black circles indicate positive correlations; red circles indicate negative correlations.

**Fig 6:**
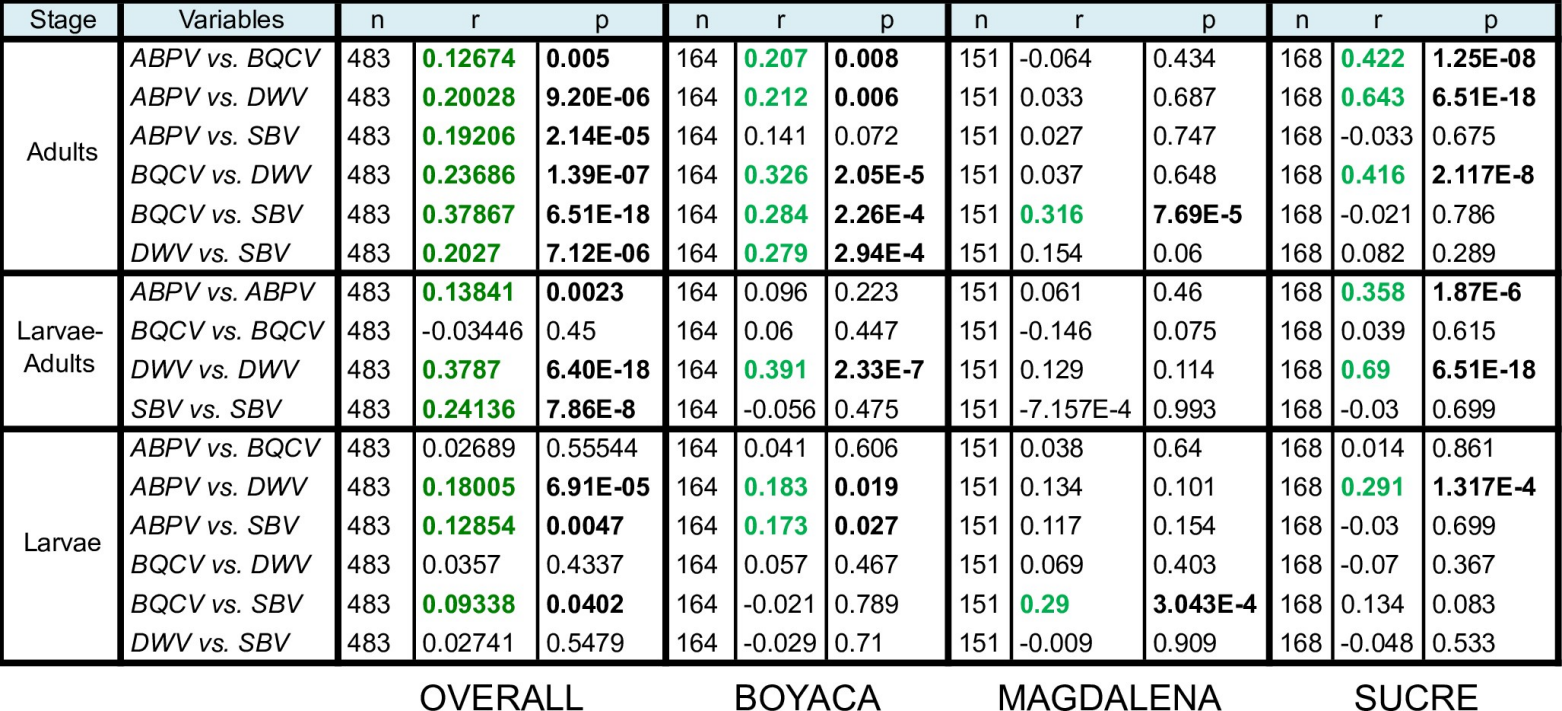
Pairwise Spearman rank correlations among viral prevalence in adults and larvae. Significant differences (p<0.05) are represented in bold case. Positive correlations are highlighted in green.

We first determined the associations concerning the prevalence of the different viruses pooled across the three regions. The overall analysis of viral prevalence between larval and adult stages for each virus, showed positive correlations for all the viruses analyzed, except for BQCV. In adults, we found significant positive correlations between each of the four viruses. In larvae, significant positive correlations were observed between ABPV and DWV, ABPV and SBV and BQCV and SBV (Figs 5 and 6).

The extent of covariation in viral prevalence varied across regions and life stages. In adults from Boyacá, all comparisons among viruses were significant with the exception of ABPV and SBV. However, in Magdalena, only BQCV and SBV were significantly correlated. In within-region analyses of larvae, correlations among viruses were less pronounced than in adults. Two of six pairs of viruses were correlated in Boyacá, and one of six pairs each in Magdalena and Sucre (Fig 6).

#### *Varroa* infestation levels

In adults of all regions combined, we found significant positive correlations between VIL and prevalence of ABPV (r = 0.106, p<0.05) and SBV (r = 0. 144, p<0.05). On the other hand, VIL did not correlate significantly with DWV or BQCV (S4 Table). In larvae, the only association observed was a negative correlation between VIL and BQCV (r = -0.115, p<0.05).

#### Altitude

We found no correlation between altitude and VIL across the three regions combined. However, in within-region analyses that removed the confounding effect of season, altitude was negatively correlated with VIL within both Boyacá (r = -0.326, p<0.001) and Magdalena (r = -0.167, p<0.05) (S4 Table). In contrast, we found positive correlations between altitude and viral prevalence for three of four viruses in adults (BQCV (r = 0.221, p<0.001) DWV (r = 0.186, p<0.001), and SBV (r = 0.146, p<0.01), and for DWV in larvae (r = 0.222, p<0.001) (S4 Table).

#### Season

Seasonal analysis was restricted to the Boyacá, the only region where samples were collected both during the rainy and dry seasons. Our results showed significant positive correlations between the rainy season and the prevalence of BQCV, ABPV, while DWV showed a positive correlation in the limit of statistical significance (r = 0.153, p = 0.05). On the other hand, in larvae, only DWV and BQCV exhibited a significant positive correlation with the rainy season (S5 Table).

## Discussion

### *Varroa* prevalence and infestation levels

Our results revealed a high *V*. *destructor* prevalence (92%). Dissemination of *V*. *destructor* has increased considerably in South America since the 1990s. This phenomenon is exemplified by the case of EHB populations in Chile, where *Varroa* prevalence increased from 80% in 2007 [59] to 93% in 2013 [45]. High percentages of *Varroa* prevalence also have been reported in AHB populations in southern Brazil (95.7%) [60], which are consistent with the prevalence observed in the present study (92%). However, *Varroa* prevalence levels have been maintained at lower levels in other countries with either predominant AHB or EHB populations, such as Uruguay (75.7%) [37] and Argentina (74%) [45], respectively. Thus, at present, there is not an evident trend between the degree of Africanization and the prevalence of *Varroa*, suggesting that other factors, including management, may modulate observed prevalence levels.

Although prevalence was high, *Varroa* infestation level (VIL) was relatively low (4.5%).

There have been few previous studies on VIL in Latin American countries. These include countries with EHB populations, such as Chile, with VIL of 5–9% [45,61], and countries with predominant AHB populations (~80% African mitotypes) such as Uruguay [62] and Mexico [63], with VIL of 7.5% [64] and 5–7.4% [65,66], respectively. Relative to these regional neighbors, Colombia appears to have the lowest VIL (4.5%), despite being the only country from this group where acaricides are not routinely used. Among these countries, Colombia also has the highest percentage of AHB (98.3%) [43]. suggesting a negative relationship between VIL and the proportion of AHBs in the population. Further studies in additional regions with comparable climates are required to confirm the generality of this trend.

For comparison, in the United States, *V*. *destructor* prevalence and infestation levels for colonies used in stationary beekeeping operations during 2009–2014 were 97.0% and 5.99% [14]. Although these values are only marginally higher compared with what we reported for Colombia, a critical difference is that AHBs in our study did not receive acaricide treatments. [67] Furthermore, it is also interesting to note that while in the United States and Canada [8,14], *V*. *destructor* is regarded as a major contributor to yearly losses that have exceeded 40% annually in the United States from 2015–2016 [68]; in Colombia, varroosis–the disease caused by *Varroa* infestation—is not considered an important problem for beekeepers and yearly colony losses were estimated at 10.8% [69].

Previous studies comparing VIL between regions with tropical and temperate climates in Mexico have found either higher VIL in tropical regions [66], or no significant differences between climates [65]. In our study, Boyacá is the region with the highest altitude, followed by Magdalena and Sucre. Thus, Boyacá has a relatively colder climate, Magdalena region, has an intermediate subtropical temperature, and Sucre a tropical climate. In our study, the overall analysis of the three regions, does not show significant correlations between VIL and altitude. However, analysis to remove regional and seasonal confounding effects revealed a significant negative correlation between VIL and altitude in the two regions with higher and more variable altitudes (Boyacá and Magdalena). In contrast, no significant correlation between VIL and altitude was found in the region with lower altitude and smaller difference in this variable (Sucre). Altogether, these results support the notion that altitude is an important factor influencing negatively VIL in tropical and neotropical regions. Thus, this effect of altitude on *Varroa* infestation resembles the reduction on *Varroa* population observed in honey bee colonies before the winter in non-temperate geographic regions.

### Viral prevalence

In colonies from the three surveyed regions, the presence of four viral pathogens was detected. However, the prevalence was lower than that reported for other countries in South America [27,45] and Mexico [66], and none of the colonies in this study showed evident symptoms of overt infection, suggesting that infection intensity was low. The Magdalena region had the highest VIL and the highest prevalence of ABPV, BQCV and SBV. In contrast, Sucre had the lowest VIL and prevalence of the four viruses. These results suggest a possible association between *V*. *destructor* infestation and viral prevalence. Our analysis shows that there is a positive correlation between VIL and the prevalence of ABPV and SBV. However, we found no significant correlations between the prevalence of VIL with DWV or with BQCV. Although this last result was unexpected, it is interesting to note that the bees in this study did not show evident symptoms of overt infection. Studies showing a positive association between DWV and VIL were conducted in colonies with higher levels of *Varroa* infestation or parasitized individuals [11,66]. It remains to be further investigated if this lack of association is related to the low VIL observed in Colombian AHBs.

The global spread of *V*. *destructor* has selected for and disseminated highly infectious and pathogenic DWV strains in European-derived populations around the world [70]. In the United States and Europe, pathogenic viruses have increased their prevalence and virulence, changing from asymptomatic to evident symptoms of infection. Comparing viral prevalence in the United States with those we are reporting as present in Colombia illustrates a sharp contrast. The reported prevalence of three viral pathogens screened in the United States during 2009–2014 was: DWV 85.09%, ABPV 21.74%, and BQCV 90.03% [14]. Interestingly, although both Colombia and the United States shared a similar VIL, the prevalence of surveyed viral pathogens in Colombia is considerably lower.

Comparison of viral prevalence between adult and larval stages could reveal potential mechanisms of viral transmission. While in adults positive correlations were found among all the viruses detected; in larvae only 50% of correlations among viruses were significant. The viruses that showed the highest prevalence (BQCV and SBV) in larvae (Fig 4) were positively correlated (Fig 6). These results suggest similar mechanisms of horizontal transmission: SBV infects both larval and adult stages, but the young larva is more susceptible to its infection [71]. Larval infection of SBV occurs via the ingestion of virus-contaminated hypopharyngeal gland secretions and pollen [19]. BQCV is one important cause of queen larvae mortality [72,73] and it is considered to be transmitted through the glandular secretions of nurses [74]. Our results suggest that BQCV affects worker larvae via a similar mechanism and that this mode of transmission could be of particular importance in AHBs inhabiting tropical regions. On the other hand, the viruses that showed lower prevalence in the larval stage (DWV and ABPV), also were positively correlated. Both DWV and ABPV showed higher prevalence in adults compared with larval stages, although this effect was only significant for ABPV. Altogether, our results are consistent with previous studies reporting that BQCV and SBV are directly transmitted via food ingestion in larvae [19,71], while ABPV and DWV are preferentially transmitted in adults through *Varroa* infestation [19,75,76].

### Altitude and seasonal weather

We found highly significant positive correlation between altitude and the prevalence of DWV, BQCV and SBV in adult bees. Our results are consistent with the finding of higher DWV prevalence in colonies from temperate climate compared with those from tropical regions in Mexico [66]. These results support the proposal that cold stress could weaken the immune response and enhance viral replication. Studies showing cold temperatures associated with down-regulation of the cellular immune response [77] and higher DWV titers [11] adds further support to this possibility.

Seasonal differences are an important factor to be considered in the study of VIL and viral prevalence. In countries with temperate climates, higher VIL and viral titers are found during the fall [14,27]. Regions near the equator do not experience substantial fluctuations in temperature. However, these tropical regions experienced important seasonal differences in pluvial precipitation and two distinct dry and rainy seasons can be distinguished during the year. One important caveat of the present study is that our experimental design was not explicitly planned to uncouple the effect of pluvial precipitation during the year. Despite this limitation, samples were collected during both the dry and rainy seasons in Boyacá, the region with the highest altitude difference. This allowed for an initial estimation of the effect of pluvial precipitations on VIL, viral prevalence and the interactions among these variables with altitude. Correlation analysis showed a significant positive association between the rainy season and viral prevalence. These results suggest that pluvial precipitations have an important effect on viral prevalence in AHB populations of tropical regions.

### Comparison between AHBs and African honey bees

Populations of American AHBs and their ancestor, the African honey bee *Apis mellifera scutellata*, seem to have developed independently adaptions to reduce *Varroa* infestation levels: While interactions between AHBs in South America and *Varroa* started in the 1980s, this parasite invaded South Africa in 1997 [78]. *A*.*m*. *scutellata* populations exhibited low *Varroa* infestation levels (<4%) and three viruses have been detected with the following prevalence: BQCV (62%), VDV-1 (also known as DWV-B) (31%) and IAPV (15%). Interestingly, despite the occurrence of these viruses, no apparent symptoms of viral diseases were noticed and, unexpectedly, DWV was not detected [38]. Thus, although African *A*.*m*. *scutellata* and South American AHBs share a considerable genetic background, they exhibit revealing similarities and differences. Both populations have low *Varroa* infestation levels and lower viral prevalence compared with EHBs, concomitant with an absence of symptoms of viral diseases. However, they apparently have a different composition of viral pathogens. Although at present VDV-1 (DWV-B) and DWV (DWV-A) are considered different viral species [79], they share 85% of identity at the nucleotide level and can establish natural recombinants [80,81]; implying that these virus isolates can be considered strains of the same viral species. Given that in *A*.*m*. *scutellata* this DWV-B strain does not appear to cause overt disease; these results are compatible with the proposal that resistance to *Varroa* is associated with a reduced selection of pathogenic strains of DWV.

### Conclusive remarks

In this report, we documented low *Varroa* infestation levels in non-acaricide treated, fully Africanized Colombian honey bee populations. These results are consistent with previous studies that found low *Varroa* infestation levels in Africanized populations of other Latin American countries and lesser infestation levels with increasing proportions of Africanized relative to European bees [45]. Moreover, despite a lack of treatment with acaricides, Colombian bees had low prevalence and exhibited no apparent symptoms of the most pervasive honey bee viruses. These findings support the view that genetic selection of local populations of honey bees is a viable strategy to manage the predominant parasites and pathogens threatening the survival of non-resistant European-derived honey bee populations. Further studies are required to better understand the interactions between *Varroa* and virus in AHBs, including the determination of viral titers and seasonal effects. This study provides valuable insights into understanding the relationship between *V*. *destructor* and the transmission and spread of honey bee viruses in AHBs.

## Supporting information

S1 Fig Histogram depicting percentages of samples with different percentages of VIL (mites per 100 adult bees). (TIF)

S1 Table Primers used for viral detection. Forward primer (F). Reverse primer (R).
(TIF)

S2 Table Percentages of viral co-infection. Percentage of samples no infected, infected with a single virus or coinfected with two, three or four of the viruses detected (DWV, SBV, BQCV and ABPV).
(TIF)

S3 Table Percentages of viral prevalence in adults and larvae per region. (TIF)

S4 Table Pairwise Spearman rank correlations among VIL, altitude and viral prevalence in adults and larvae. Significant correlations (p<0.05) are represented in bold case. Positive and negative correlations are highlighted in green and red, respectively.
(TIF)

S5 Table Pairwise Spearman rank correlations between seasonal weather (rainy season) and viral prevalence in adults (A) and larvae (L). Significant correlations (p<0.05) are represented in bold case. Positive and negative correlations are highlighted in green and red, respectively.
(TIF)

S1 Dataset (XLSX)

## References

[1] Kent R. The Introduction and Diffusion of the African Honeybee in South America. Yearbook of the Association of Pacific Coast Geographers. 50: University of Hawaii Press; 1988. p. 21–43.

[2] Caron D. Africanized honey bees in the Americas. Medina Ohio: A.I. Rood Co; 2001.

[3] Kerr W. Introduction of african honey bees in Brazil. Brasil Apicola. 1957;3(211–213).

[4] Martin S, Medina LM. Africanized honeybees have unique tolerance to *Varroa* mites. Trends Parasitol. 2004;20(3):112–4. 10.1016/j.pt.2004.01.001 15036029

[5] Guzman-Novoa E, Sánchez A., Page RE, García T. Susceptibility of European and Africanized honeybees (*Apis mellifer*a L.) and their hybrids to *Varroa jacobsoni* Oud. Apidologie. 1996;27:93–103.

[6] Rosenkranz P. Honey bee (*Apis mellifera* L.) tolerance to *Varroa jacobsoni* Oud. in South America. Apidology. 1999;30:159–72.

[7] Neumann P, Carreck NL. Honey bee colony losses. J Apic Res. 2010;49(1):1–6.

[8] Guzmán-Novoa E, Eccles L, Calvete Y, Mcgowan J, Kelly PG, Correa-Benitez A. *Varroa destructor* is the main culprit for the death and reduced populations of overwintered honey bee (*Apis mellifera*) colonies in Ontario, Canada. Apidologie. 2010;41(4):443–50.

[9] Martin S, Highfield AC, Brettell L, Villalobos EM, Budge GE, et al. Global honey bee viral landscape altered by a parasitic mite. Science. 2012;336(6086):1304–6. 10.1126/science.1220941 22679096

[10] Bowen-Walker P, Martin SJ, Gunn A. The transmission of deformed wing virus between honeybees (*Apis mellifera* L) by the ectoparasitic mite *Varroa jacobsoni* Oud. J Invertebr Pathol. 1999;73:101–6. 10.1006/jipa.1998.4807 9878295

[11] Di Prisco G, Zhang X, Pennacchio F, Caprio E, Li J, et al. Dynamics of persistent and acute deformed wing virus infections in honey bees, Apis mellifera. Viruses. 2011;3:2425–41. 10.3390/v3122425 22355447 PMC3280512

[12] Möckel N, Gisder S, Genersch E. Horizontal transmission of deformed wing virus: pathological consequences in adult bees (*Apis mellifera*) depend on the transmission route. J Gen Virol. 2011;92(2):370–7. 10.1099/vir.0.025940-0 20965988

[13] Dainat B, Neumann P. Clinical signs of deformed wing virus infection are predictive markers for honey bee colony losses. J Invertebr Pathol. 2013;112(3):278–80. 10.1016/j.jip.2012.12.009 23270875

[14] Traynor K, Rennich K, Forsgren E, Rose R, Pettis J et al. Multiyear survey targeting disease incidence in US honey bees. Apidologie. 2016;47:325–47.

[15] Gisder S, Genersch E. Special Issue: Honey Bee Viruses. Viruses. 2015;7(10):2885. 10.3390/v7102885 26702462 PMC4632393

[16] Chen Y, Pettis JS, Collins A, Feldlaufer MF. Prevalence and transmission of honey bee viruses. Appli Environ Microbiol. 2006;72:606–11. 10.1128/AEM.72.1.606-611.2006 PMC1352288 16391097

[17] Cornman R, Tarpy DR, Chen Y, Jeffreys L, Lopez D, et al. Pathogen webs in collapsing honey bee colonies. PLoS One. 2012;7(8):e43562. 10.1371/journal.pone.0043562 22927991 PMC3424165

[18] Brutscher L, McMenamin AJ, Flenniken ML. The Buzz about Honey Bee Viruses. PLoS Pathog. 2016;12(8):e1005757. 10.1371/journal.ppat.1005757 27537076 PMC4990335

[19] Chen Y, Siede R. Honey Bee Viruses. Adv Virus Res. 2007;70:33–80. 10.1016/S0065-3527(07)70002-7 17765703

[20] Antúnez K, D’Alessandro B, Corbella E, Zunino P. Detection of chronic bee paralysis virus and acute bee paralysis virus in Uruguayan honeybees. J Invertebr Pathol. 2005;90(1):69–72. 10.1016/j.jip.2005.07.001 16169006

[21] Teixeira E, Chen Y, Message D, Pettis J, Evans JD. Virus infections in Brazilian honey bees. J Invertebr Pathol. 2008;99(1):117–9. 10.1016/j.jip.2008.03.014 18471826

[22] Teixeira E, Chen YP, Message D, Boncristiani HF, Pettis JS, Evans JD. Israeli acute paralysis virus in Africanized honey bees in southeastern Brazilian Apiaries. J Apic Res. 2012;51(3):282–4.

[23] Reynaldi F, Sguazza GH, Pecoraro MR, Tizzano MA, Galosi CM. First report of viral infections that affect argentine honeybees. Environ Microbiol Rep. 2010;2(6):749–51. 10.1111/j.1758-2229.2010.00173.x 23766280

[24] Reynaldi F, Sguazza GH, Tizzano MA, Fuentealba N, Galosi CM, Pecoraro MR. First report of Israeli acute paralysis virus in asymptomatic hives of Argentina. Rev Argent Microbiol. 2011;43(2):84–6. 10.1590/S0325-75412011000200003 21731968

[25] Freiberg M, De Jong D, Message D, Cox-Foster D. First report of sacbrood virus in honey bee (*Apis mellifera*) colonies in Brazil. Genet Mol Res. 2012;11(3):3310–4. 10.4238/2012.September.12.14 23079825

[26] Rodríguez M, Vargas M, Antúnez K, Gerding M, Ovídio Castro F, Zapata N. Prevalence and phylogenetic analysis of honey bee viruses in the Biobío Region of Chile and their association with other honey bee pathogens. Chilean journal of agricultural research. Chilean J Agric Res. 2014;74(2):170–7.

[27] Antúnez K, Anido M, Branchiccela B, Harriet J, Campa J, et al. Seasonal variation of honeybee pathogens and its association with pollen diversity in Uruguay. Microb Ecol. 2015;70(2):522–33. 10.1007/s00248-015-0594-7 25794593

[28] Genersch E, Aubert M. Emerging and re-emerging viruses of the honey bee (*Apis mellifera* L.). Vet Res. 2010;41(6):54. 10.1051/vetres/2010027 20423694 PMC2883145

[29] Yue C, Schroeder M, Gisder S, Genersch E. Vertical-transmission routes for deformed wing virus of honeybees (*Apis mellifera*). J Gen Virol. 2007;88:2329–36. 10.1099/vir.0.83101-0 17622639

[30] de Miranda J, Fries I. Venereal and vertical transmission of deformed wing virus in honeybees (A*pis mellifera* L). J Invertebr Pathol. 2008;98:184–9. 10.1016/j.jip.2008.02.004 18358488

[31] Chen Y, Pettis JS, Collins A, Feldlaufer MF. Prevalence and transmission of honeybee viruses. Appl Environ Microbiol. 2006;72:606–11. 10.1128/AEM.72.1.606-611.2006 16391097 PMC1352288

[32] Martin S. The role of *Varroa* and viral pathogens in the collapse of honeybee colonies: a modelling approach. J Appl Ecol. 2001; 38:1082.

[33] Sumpter D, Martin SJ. The dynamics of virus epidemics in *Varroa* infested honey bee colonies. J Anim Ecol. 2004;73:51–63.

[34] Emsen B, Hamiduzzaman MM, Goodwin PH, Guzman-Novoa E. Lower virus infections in Varroa destructor-infested and uninfested brood and adult honey bees (*Apis mellifera*) of a low mite population growth colony compared to a high mite population growth colony. PLoS One. 2015;10(2):e0118885. 10.1371/journal.pone.0118885 25723540 PMC4344307

[35] Rosenkranz P, Aumeier P, Ziegelmann B. Biology and control of *Varroa destructor*. J Invertebr Pathol. 2010;103(1):S96–119. 10.1016/j.jip.2009.07.016 19909970

[36] Junkes L, Guerra Júnior JCV, Moretto G. Varroa destructor mite mortality rate according to the amount of worker broods in Africanized honey bee (*Apis mellifera* L.) colonies. Acta Sci Biol Sci. 2007;29:305–8.

[37] Anido M, Branchiccela B, Castelli L, Harriet J, Campá J, Zunino P, et al. Prevalence and distribution of honey bee pathogens in Uruguay. J Apic Res. 2015;54:532–40.

[38] Strauss U, Human H, Gauthier L, Crewe RM, Dietemann V, Pirk CW. Seasonal prevalence of pathogens and parasites in the savannah honeybee (*Apis mellifera scutellata*). J Invertebr Pathol. 2013;114(1):45–52. 10.1016/j.jip.2013.05.003 23702244

[39] Moretto G, Gonçalves LS, De Jong D, Bichuette MZ. The effects of climate and bee race on *Varroa jacobson*i Oud infestations in Brazil. Apidologie. 1991;22:197–203.

[40] Guzman-Novoa E, Vandame R, Arechavaleta ME. Susceptibility of European and Africanized honey bees (*Apis mellifera* L.) to Varroa jacobsoni Oud. in Mexico. Apidologie. 1999;30:173–82.

[41] Medina-Flores C, Guzman-Novoa E, Hamiduzzaman MM, Aréchiga-Flores CF, López-Carlos MA. Africanized honey bees (*Apis mellifera*) have low infestation levels of the mite *Varroa destructor* in different ecological regions in Mexico. Genet Mol Res. 2014;13:7282–93. 10.4238/2014.February.21.10 24634296

[42] Arechavaleta-Velasco M, Guzman-Novoa E. Producción de miel de colonias de abejas (Apis mellifera L.) tratadas y no tratadas con fluvalinato contra *Varroa jacobsoni* Oudemans en Valle de Bravo, Estado de Mexico. Vet Méx. 2000;31(4):381–4.

[43] Tibata V, Arias E, Corona M, Ariza-Botero F, Figueroa-Ramírez J and Junca H. Determination of the Africanized mitotypes in populations of honey bees (Apis mellifera L.) of Colombia. J Apic Res. 2017;57:219–27.

[44] Hamiduzzaman M, Guzman-Novoa E, Goodwin PH, Reyes-Quintana M, Koleoglu G et al. Differential responses of Africanized and European honey bees (Apis mellifera) to viral replication following mechanical transmission or *Varroa destructo*r parasitism. J Invertebr Pathol. 2015;126:12–20. 10.1016/j.jip.2014.12.004 25527405

[45] Maggi M, Antúnez K, Invernizzi C, Aldea P, Vargas M, Negri P, et al. Honeybee health in South America. Apidologie. 2016;47(6):835.

[46] Knoben W, Woods RA, Freer JE. Global bimodal precipitation seasonality: A systematic overview. Int J Climatol. 2019;39:558–67.

[47] De Jong D, Roma DD, Goncalves LS. A comparative analysis of shaking solutions for the detection of *Varroa jacobsoni* on adult honeybees. Apidologie. 1982;13:297–306.

[48] Chen Y, Higgins JA, Feldlaufer MF. Quantitative real-time reverse transcription-PCR analysis of deformed wing virus infection in the honeybee (Apis mellifera L.). Appl Environ Microbiol. 2005;71(1):436–41. 10.1128/AEM.71.1.436-441.2005 15640219 PMC544241

[49] Tentcheva D, Gauthier L, Zappulla N, Dainat B, Cousserans F, et al. Prevalence and seasonal variations of six bee viruses in Apis mellifera L. and *Varroa destructor* mite populations in France. Appl Environ Microbiol. 2004;70(12):7185–91. 10.1128/AEM.70.12.7185-7191.2004 15574916 PMC535170

[50] Kukielka D, Esperón F, Higes M, Sánchez-Vizcaíno JM. A sensitive one-step real-time RT-PCR method for detection of deformed wing virus and black queen cell virus in honeybee Apis mellifera. J Virol Methods. 2008;147(2):275–81. 10.1016/j.jviromet.2007.09.008 17964669

[51] Kukielka D, Sánchez-Vizcaíno JM. One-step real-time quantitative PCR assays for the detection and field study of Sacbrood honeybee and Acute bee paralysis viruses. J Virol Methods. 2009; 161(2):240–6. 10.1016/j.jviromet.2009.06.014 19559729

[52] Ribière M, Triboulot C, Mathieu L, Aurières C, Faucon JP, Pépin M. Molecular diagnosis of chronic bee paralysis virus infection. Apidologie. 2002;33(3):339–52.

[53] Maori E, Tanne E, Sela I. Reciprocal sequence exchange between non-retro viruses and hosts leading to the appearance of new host phenotypes. Virology. 2007;362(2):342–9. 10.1016/j.virol.2006.11.038 17275871

[54] Stoltz D, Shen XR, Boggis C, Sisson G. Molecular diagnosis of Kashmir bee virus infection. J Apic Res 1995;34(3):153–60.

[55] Chen Y, Pettis JS, Feldlaufer MF. Detection of multiple viruses in queens of the honey bee Apis mellifera L. J Invertebr Pathol. 2005;90(2):118–21. 10.1016/j.jip.2005.08.005 16214161

[56] Fox J, Weisberg S. An {R} Companion to Applied Regression. Second Edition ed. Thousand Oaks CA: Sage; 2011.

[57] de Mendiburu F. Agricolae: Statistical Procedures for Agricultural Research. R package version 1.2–4. 2016.

[58] RStudio Team. RStudio: Integrated Development for R. Boston, MA: RStudio Inc; 2016.

[59] Neira M, Dussaubat C, Manquián N, Bahamonde P, Vera M. Sanidad apícola en Chile, situación de las principales enfermedades. Agro Sur. 2007;35(1):47–8.

[60] Diniz N, Soares AEE, Sheppard WS, Del Lama MA. Genetic structure of honeybee populations from southern Brazil and Uruguay. Genet Mol Biol. 2003;26(1):47–52.

[61] Aldea P, Rodríguez R, Olivares A, Farfán M, Riveros D, Núñez F, et al. Effect of ambient temperature and humidity conditions on the efficacy of organic treatments against *Varroa destructor* in differ- ent climatic zones of Chile. J Agric Sci Technol. 2013;A 3:474–83.

[62] Branchiccela B, Aguirre C, Parra G, Estay P, Zunino P, Antúnez K. Genetic changes in Apis mellifera after 40 years of Africanization. Apidologie. 2014;45:752–6.

[63] Kraus F, Franck P, Vandame R. Asymmetric introgression of African genes in honeybee populations (Apis mellifera L.) in Central Mexico. Heredity. Heredity. 2007;99(2):233‐40. 10.1038/sj.hdy.6800988 17473860

[64] Invernizzi CA, Antúnes K. Campa J. Harriet J. Mendoza Y. Santos E. Zunino P. Situación sanitaria de las abejas melíferas en Uruguay. Veterinaria. 2011;47(181):15–27.

[65] Tapia-Gonzalez J, Alcazar-Oceguera G, Macías-Macías JO, Contreras-Escareño F, Tapia-Rivera JC, Petukhova T, et al. Varroosis in honey bees in different environmental and regional conditions of Jalisco, Mexico. Ecosist Recur Agropec. 2019;6(17):243–51.

[66] Anguiano-Baez R, Guzman-Novoa E, Md Hamiduzzaman M, Espinosa-Montaño LG, Correa-Benítez A. Varroa destructor (Mesostigmata: Varroidae) Parasitism and Climate Differentially Influence the Prevalence, Levels, and Overt Infections of Deformed Wing Virus in Honey Bees (Hymenoptera: Apidae). J Insect Sci. 2016;16(1):44. 10.1093/jisesa/iew029 27252482 PMC4887826

[67] Judice C, Hartfelder K., Pereira G. A. G. Caste-specific gene expression in the stingless bee *Melipona quadrifasciat*a—Are there common patterns in highly eusocial bees? Insectes Sociaux. 2004;51(4):352–8.

[68] Kulhanek K, Steinhauer N, Rennich K, Caron DM, Sagili RR, et al. A national survey of managed honey bee 2015–2016 annual colony losses in the USA. J Apic Res. 2017;56(4):328–40.

[69] Requier F, Antúnez K, Morales CL, Patricia Aldea P, Castilhos D, et al. Trends in beekeeping and honey bee colony losses in Latin America. J Apic Res. 2018. 10.1080/00218839.2018.1494919

[70] Wilfert L, Long G, Leggett HC, Schmid-Hempel P, Butlin R, Martin SJ, et al. Deformed wing virus is a recent global epidemic in honeybees driven by *Varroa* mites. Science. 2016;351(6273):594–7. 10.1126/science.aac9976 26912700

[71] Ball B, Bailey L. Viruses. Honey Bee Pest, Predators, & Diseases. Medina, OH.: The A. I. Root Co.; 1997. p. 11–31.

[72] Bailey L, Woods RD. Two more small RNA viruses from honey bees and further observations on sacbrood and acute bee-paralysis viruses. J Gen Virol. 1977;37:175–82.

[73] Anderson D. Pathogen and queen bees. Australasian Beekeeper 1993;94:292–6.

[74] Bailey L. Viruses of honeybees. Bee World. 1982;63:165–73.

[75] Ball B, Allen MF. The prevalence of pathogens in honey bee (Apis mellifera) colonies infested with the parasitic mite *Varroa jacobsoni*. Ann Appl Biol. 1988;113:237–44.

[76] Ball B. *Varroa jacobsoni* as a virus vector. In: Cavalloro R, editor. Present Status of Varroatosis in Europe and Progress in the *Varroa* Mite Control. Luxembourg: EEC-EAEC; 1989. p. 241–4.

[77] Steinmann N, Corona M, Neumann P, Dainat B. Overwintering Is Associated with Reduced Expression of Immune Genes and Higher Susceptibility to Virus Infection in Honey Bees. PLoS One. 2015;10(6):e0129956. 10.1371/journal.pone.0129956 26121358 PMC4486728

[78] Allsopp M. Varroa jacobsoni in South Africa. S Afr Bee J. 1997; 69:73–82.

[79] Valles S, Chen Y, Firth AE, Guérin DMA, Hashimoto Y, Herrero S, et al. ICTV Virus Taxonomy Profile: Iflaviridae. J Gen Virol. 2017;98(4):527–8. 10.1099/jgv.0.000757 28382900 PMC5657024

[80] Moore J, Jironkin A, Chandler D, Burroughs N, Evans DJ, Ryabov EV. Recombinants between Deformed wing virus and *Varroa destructor* virus-1 may prevail in Varroa destructor-infested honeybee colonies. J Gen Virol. 2011;92(1):156–61. 10.1099/vir.0.025965-0 20926636

[81] Zioni N, Soroker V, Chejanovsky N. Replication of *Varroa destructor* virus 1 (VDV-1) and a Varroa destructor virus 1-deformed wing virus recombinant (VDV-1-DWV) in the head of the honey bee. Virology. 2011;417(1):106–12. 10.1016/j.virol.2011.05.009 21652054

