# Supplementary figures and images for: Africanized honey bees in Colombia exhibit high prevalence but low level of infestation of Varroa mites and low prevalence of pathogenic viruses
Source: PLoS One. 2021 May 20;16(5):e0244906. doi: 10.1371/journal.pone.0244906 (PMC8136659; doi:10.1371/journal.pone.0244906)

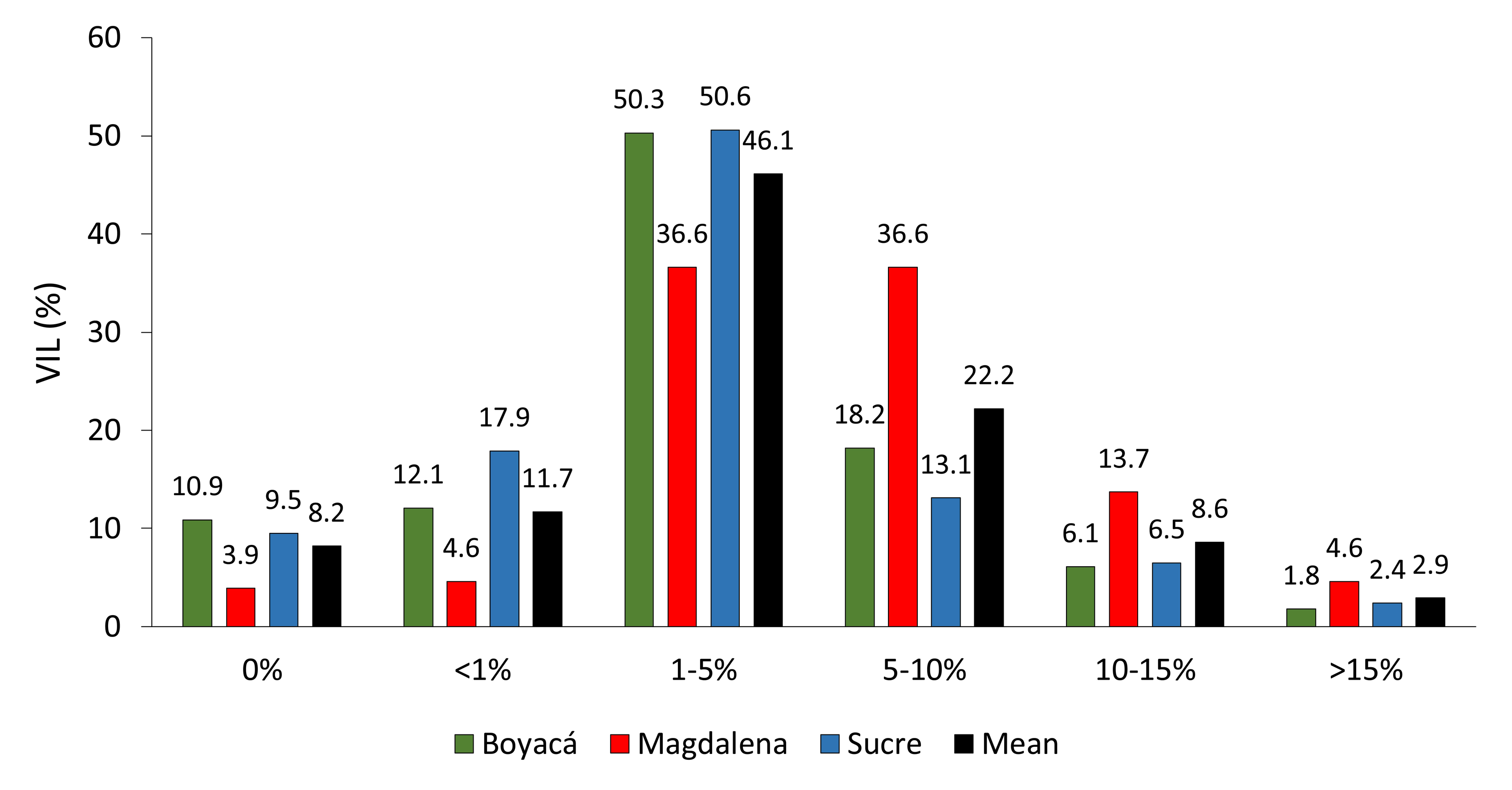

Supplement: S1 Fig — (TIF) [file pone.0244906.s001.tif]

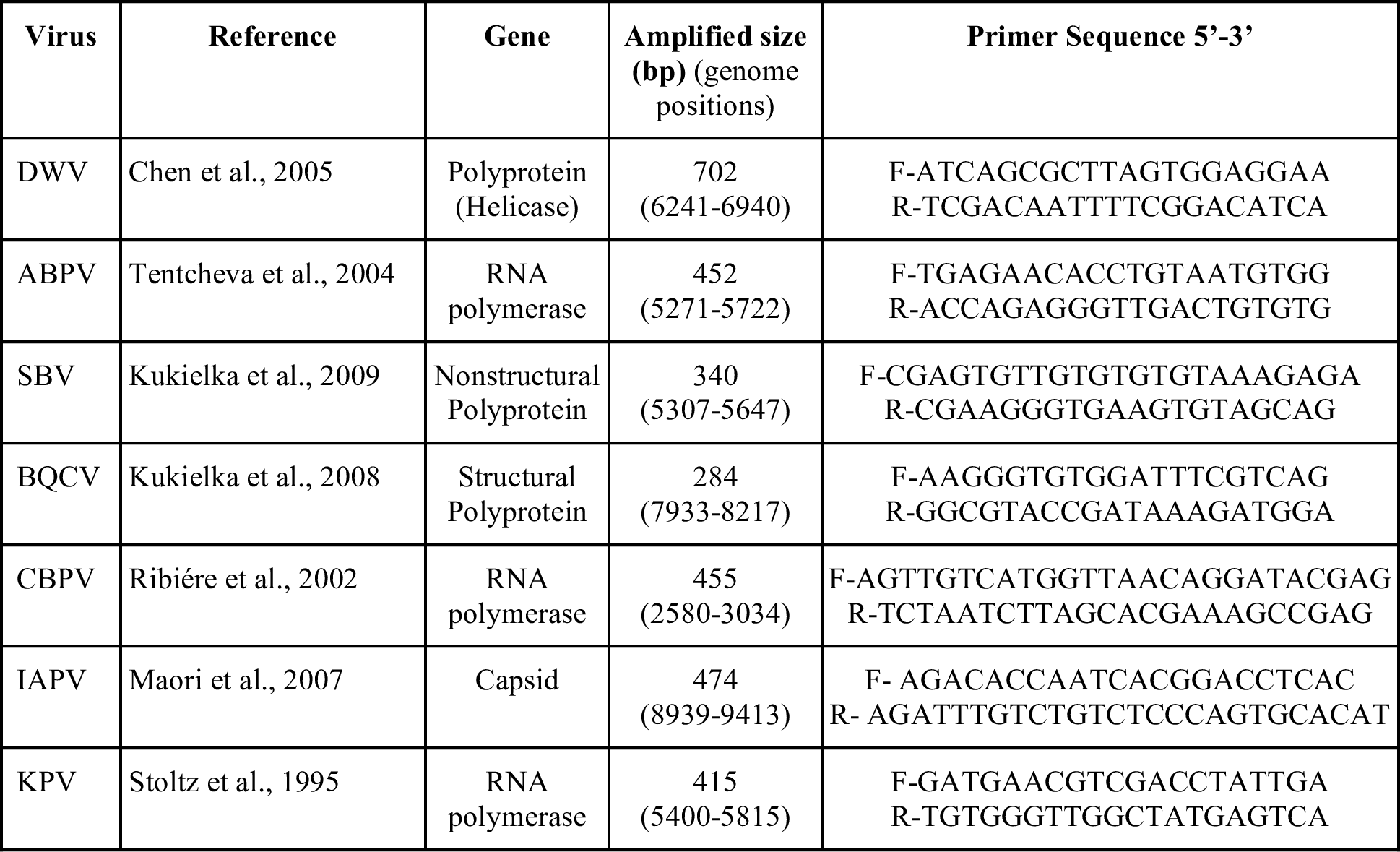

Supplement: S1 Table — Forward primer (F). Reverse primer (R). (TIF) [file pone.0244906.s002.tif]

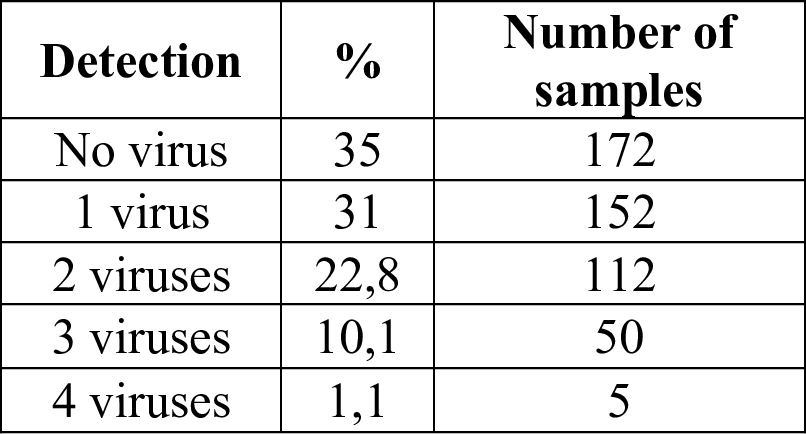

Supplement: S2 Table — Percentage of samples no infected, infected with a single virus or coinfected with two, three or four of the viruses detected (DWV, SBV, BQCV and ABPV). (TIF) [file pone.0244906.s003.tif]

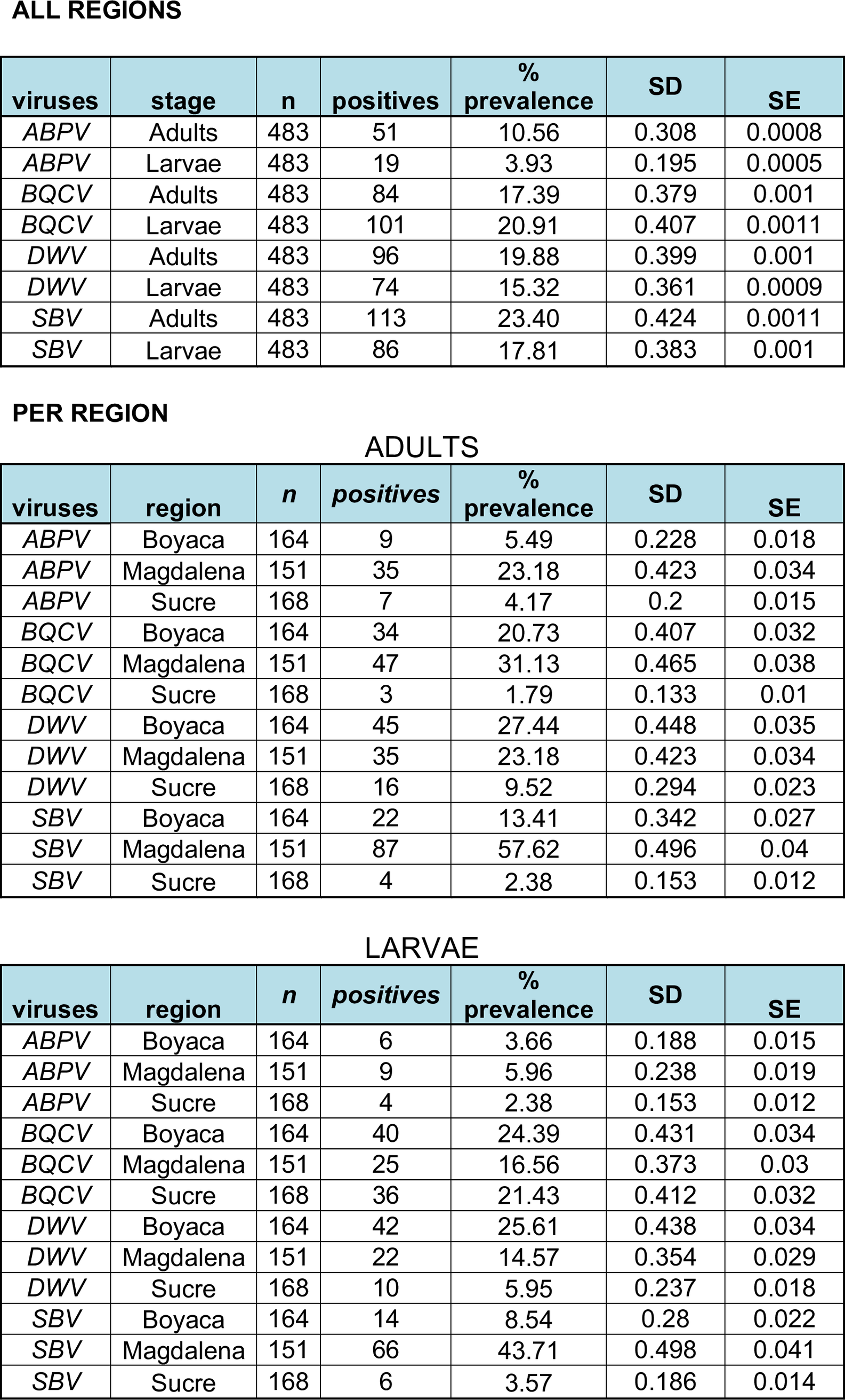

Supplement: S3 Table — (TIF) [file pone.0244906.s004.tif]

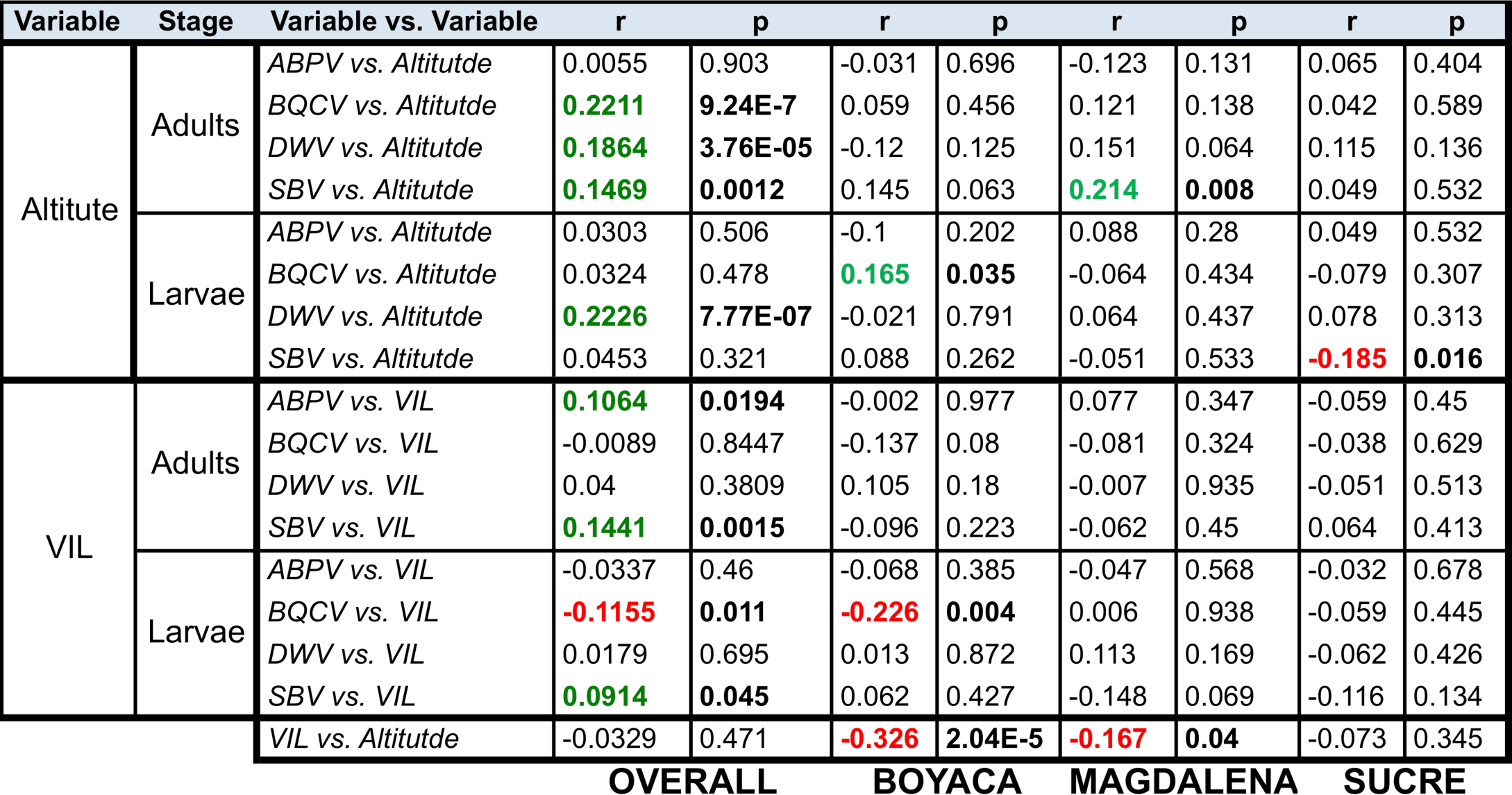

Supplement: S4 Table — Significant correlations (p<0.05) are represented in bold case. Positive and negative correlations are highlighted in green and red, respectively. (TIF) [file pone.0244906.s005.tif]

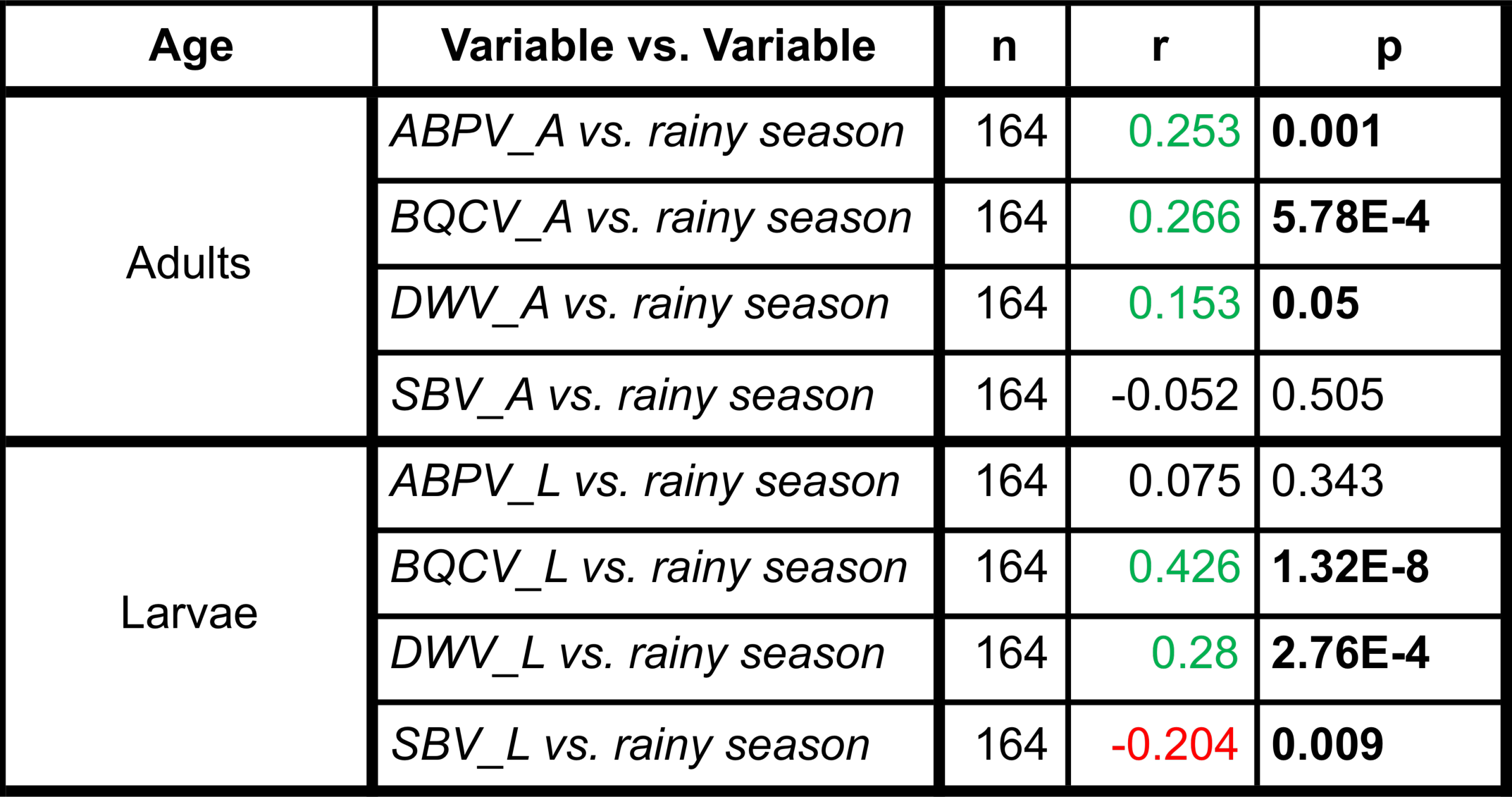

Supplement: S5 Table — Significant correlations (p<0.05) are represented in bold case. Positive and negative correlations are highlighted in green and red, respectively. (TIF) [file pone.0244906.s006.tif]
